# Application of Low-Intensity Modified Constraint-Induced Movement Therapy to Improve the Affected Upper Limb Functionality in Infantile Hemiplegia with Moderate Manual Ability: Case Series

**DOI:** 10.3390/children7090127

**Published:** 2020-09-04

**Authors:** Rocío Palomo-Carrión, Rita-Pilar Romero-Galisteo, Elena Pinero-Pinto, Purificación López-Muñoz, Helena Romay-Barrero, Francisco García-Muro San José

**Affiliations:** 1 Department of Nursery, Physiotherapy and Occupational Therapy, Faculty of Physiotherapy, University of Castilla-La Mancha, 13001 Ciudad Real, Spain; rocio.palomo@uclm.es (R.P.-C.); purificacion.lopez@uclm.es (P.L.-M.); helena.romay@uclm.es (H.R.-B.); 2 Department of Physiotherapy, Faculty of Science Health, University of Málaga, 29016 Málaga, Spain; 3 Department of Physical Therapy, Faculty of Nursery, Physiotherapy and Podiatry, University of Seville, 41004 Sevilla, Spain; 4 Department of Physiotherapy, Faculty of Medicine, University San Pablo-CEU, 28003 Madrid, Spain; fgarciamuro@ceu.es

**Keywords:** family, infantile hemiplegia, modified Constraint-Induced Movement Therapy, physical therapy modalities, upper extremity

## Abstract

Objective: To assess the functionality of the affected upper limb in children diagnosed with hemiplegia aged between 4 and 8 years after applying low-intensity modified Constraint-Induced Movement Therapy (mCIMT). Methods: Prospective case series study. A mCIMT protocol was applied for five weeks, with two hours of containment per day. The study variables were quality of movement of the upper limb, spontaneous use, participation of the affected upper limb in activities of daily living, dynamic joint position, grasp–release action, grasp strength, supination and extension elbow movements. Four measurements were performed, using the quality of upper extremity test (QUEST) scale, the Shriners Hospital for Children Upper Extremity Evaluation (SHUEE) Evaluation, a hand dynamometer and a goniometer. Results: The sample was composed of eight children with moderate manual ability. Statistically significant differences were detected in all the studied variables (*p* < 0.05) between the pre-treatment and post–treatment results (Week 0–Week 5), except for upper limb dressing, putting on splints and buttoning up. In the first week, the changes were statistically significant, except for protective extension, grasp strength, grasp–release and all functional variables (level of functionality and participation of the patient’s upper limbs) in the SHUEE Evaluation (*p* > 0.05). The greatest increase occurred in spontaneous use from Assessment 1 to Assessment 4 (*p* = 0.01), reaching 88.87% active participation in bimanual tasks. The quality of movement of the upper limb exhibited a significant value due to the increase in dissociated movements and grasp (*p* = 0.01). Conclusion: A low dose (50 h) of mCIMT increased the functionality of children diagnosed with congenital hemiplegia between 4 and 8 years of age with moderate manual ability.

## 1. Introduction

Infantile hemiplegia is a subtype of infantile cerebral palsy, characterized by the affectation of one of the hemibodies as a consequence of brain injury. Its prevalence is 1 case per 1300 live births [1]. There is more affectation of the upper limb than the lower limb due to the alteration of the corticospinal tract. The affected hand has a deficit in proprioception and tactile perception, which hinders fine motor skills, generally those of the fingers and the strength exerted by them [2]. Sensory abnormalities, weak grasp and loss of manual ability (fine movements) may appear, specifically in the fingers, with slower movements, poorer coordination and longer phases associated with mirror movements. This leads to a decrease in the use of the affected hand and often interferes with the manual ability of the healthy upper limb [3].

From early childhood, children with hemiplegia, even the least affected, use their healthy hand as the dominant hand in all tasks. Therefore, they learn “not to use” their affected upper limb, which is known as developmental disregard [4]. This “non-use” of the affected upper limb produces an increase in muscle tone in the affected segment, poor motor control, decreased active and passive range of motion, generalized weakness and delayed musculoskeletal maturation. The non-use affectation is caused by neural dysfunction as a result of brain injury. This neuronal alteration [5] can be improved through the activation of certain brain areas that remained inactive after the brain lesion and also through experience and learning (trial–error).

Thus, in order to improve the affected upper limb “non-use”, Constraint-Induced Movement Therapy (CIMT) is used [6,7], which consists of constraining the healthy upper limb with a whole or partial containment (glove), thus promoting the use of the affected upper limb in activities of daily living. The programmed tasks integrate the repetition of the motor action with a variety of exercises. The use of CIMT has spread in recent years among physiotherapists and occupational therapists, due to the large number of studies that support the effectiveness of this intervention compared to traditional interventions that do not restrict the use of the healthy side [8]. However, in its original conception, two premises had to be met: the restriction of the less-affected upper limb and the application of an intensive treatment applied in a structured way to the upper limbs [9]. Different variants of CIMT have emerged over the years, under the term “modified Constraint-Induced Movement Therapy” (mCIMT). Likewise, there are a variety of protocols used for CIMT, in which different containment systems are proposed [10,11,12,13,14,15] in which it is the dose that varies [6,12,14,16,17,18,19,20,21,22,23,24,25,26,27]. In this line, one of the modifications widely used in pediatrics is the one based on mCIMT, which constrains the healthy upper limb for less than 3 h [23,28,29,30]. McConnell et al. [31] found that a less intensive treatment (63 h of treatment over 21 days) produced similar benefits compared to a more intensive approach (126 h of treatment over 21 days). Functional gains may be feasible for some children with a less intense program adjusted to 20 h of therapy in more than two consecutive weeks [31]. According to Schweighofer et al. [32], the existence of a “functional threshold” would be necessary for the maintenance of functionality after therapy, below which the use of the upper limb decreases while the benefits to the individual remain above such threshold. It would be useful to determine the specific doses of therapy in each patient.

McConnell et al. [31] applied mCIMT for 2 weeks with two hours of dose per day in a clinical setting, designed for children aged 8–15 years, and the therapist increased the dose to continue with the use of the affected upper limb for 30 min at home. The children executed 20 h of total dose with functional changes. We proposed to apply 50 h of total dose, with the same distribution per day according to this study, although increasing the dose by 30 h, since the participants in our study were younger than those in McConnell et al. [31] and the therapy was performed at home, thus the children and their families needed more time to obtain significant results. Thus, we decided to assess the children at Week 2 of treatment (20 h) in order to verify whether the changes would be the same at Week 5 (after treatment with a total dose of 50 h). The systematic review [33] included 31 papers, each of which applied different doses per day, total doses, measurement tools, etc. All 31 studies were compared, with the main difficulty being that the children had different manual ability levels, and it would be ideal to know the correct dose for each level. In this systematic review, the manual ability levels were assessed with the Manual Classification System, MACS; some studies show Levels I–III, I–IV or I–V [34]. We could consider a moderate hand ability level for children classified as Level I–II in MACS. These levels reflect that the children are independent in the execution of activities using one or two hands or including compensation strategies (neck, trunk, mouth, etc.) to complete the bimanual task; they also show that the movement restrictions do not impede the complete use of the affected upper limb.

However, most of the published studies include functional activities in their treatment proposals. To our knowledge, a few of them contemplate an ecological vision of human development as initially proposed by Bronfenbrenner [35]. According to this perspective, it is crucial to incorporate the principles of therapy to the environment in which the child develops, which is essential to ensure the long-term persistence of the achieved results [36]. From the ecological point of view, the evolution of the child is understood as a process of progressive differentiation of the activities that he/she carries out, his/her role and his/her interactions with the environment. The interactions and transactions established between the child and the elements of his/her environment are very important, especially with his/her parents.

One of the latest reviews on the use of CIMT [8] showed that the risk of bias of the analyzed clinical trials was between moderate and high; therefore, a new randomized controlled trial is proposed in this work, whose main objective was to analyze the effectiveness of the use of CIMT by modifying the applied dose. Moreover, the ecological perspective of development was considered, introducing functional tasks that children usually carry out in their usual environment. To our knowledge, no study has been published in Spain that combines a low dose of treatment with the performance of functional activities at home with the parents. For this reason, we consider assessing the functionality of the affected upper limb in children diagnosed with congenital hemiplegia with moderate manual ability between 4 and 8 years of age after applying low-intensity modified constraint-induced movement therapy (50 h) at home

## 2. Materials and Methods

This is a case series, prospective and longitudinal study with non-probability sampling (clinical.gov NCT02178371). The study was approved (060-13) by the ethics committee of the CEU-San Pablo University of Madrid in accordance with the Declaration of Helsinki of the World Medical Association. Before initiating the study, an informed consent form was given to the children’s families to participate, which guaranteed the right to withdraw from the study at any time, if required by the participants.

The inclusion criteria were a medical diagnosis of left/right congenital infantile hemiplegia, age between 4 and 8 years, lack of activity of the affected upper limb, ability to exceed 10° extension in the metacarpophalangeal and interphalangeal joint, ability to complete a 20° extension of the wrist of the affected upper limb, adequate cognitive development to understand the verbal orders given for the execution of tasks and cooperation in their execution. In the same way, the exclusion criteria were visual problems that prevented the individual from carrying out the intervention, suffering from significant balance disturbances that put the child at risk of falling as a consequence of having the healthy upper limb contained, presenting uncontrolled epilepsy and having received botulinum toxin within 6 months prior to the intervention.

All the children were selected according to the inclusion criteria by their rehabilitation doctor of the “Virgen de la Salud” Hospital in Toledo for the execution of the therapy.

### 2.1. Intervention Method

The study was carried out over a period of 5 weeks of treatment, containing the healthy upper limb for 2 h per day [11,13] (not continuously) from Monday to Friday. McConnell et al. [31] and Al-Oraibi 2011 [21] used mCIMT for 2 h per day, obtaining positive results, and the children wore the hand containment for 96% of the total dose [13]. Thus, in our study, the children were requested to perform the structured activities for two non-consecutive hours, with the aim of increasing the adherence and avoiding great physical effort. The tasks were separated by at least 30 min of rest to allow the children to concentrate on the next activities, perform properly and stay motivated. The families were advised to set aside one hour in the early afternoon and another hour in the late afternoon to ensure that the child was attentive, frustration-free and effort-tolerant. The families were also instructed to run a full hour, repeating the activity and designing a story, in which the child was the protagonist and the activity was an enjoyable game to complete. Each of the proposed hours could be divided into periods of 30 min to improve the child’s attention and motivation in case the child was tired during the treatment. We chose this minimum period of time, since it is the smallest dose of mCIMT used to treat babies aged 3 to 8 months with babyCIMT, in order to ensure sustained attention to activity [37]. This minimum dose of 30 min was used also in a systematic review [11]. When dealing with children from 4 to 8 years old, attention is greater and maintained for a longer time, which allows establishing a continuity of 1 h of intervention. The intervention was carried out by the family at home. Previously, an informative meeting was held with the parents, in which all the details of the study were explained to them and a program of unimanual activities was given to them to be executed with the affected upper limb every treatment week (Table 1). The activities were programmed to work different movements that were limited in the affected upper limb: shoulder flexion, elbow extension, forearm supination, wrist extension and grasp. Each activity was repeated for around 15 min to obtain a lesson about a functional strategy to use in their usual activities. Each intervention hour was completed with four activities (Table 1), which were repeated in the second hour to increase the movement learning and the possibility to modify and obtain new functional strategies to perform the activity. The same activities were performed every week (first and second hours from Monday to Friday) to improve the movement (shoulder flexion, forearm supination, elbow extension, wrist extension and grasp–release).

The parents were instructed to correctly carry out the intervention avoiding possible errors during the treatment protocol. The weekly exercises, the containment technique and its correct use were also taught to them. The treatment was only initiated when the families and children were confident about it. The family and therapist met every week to assess the activities and make adjustments if necessary.

**Figure 1:**
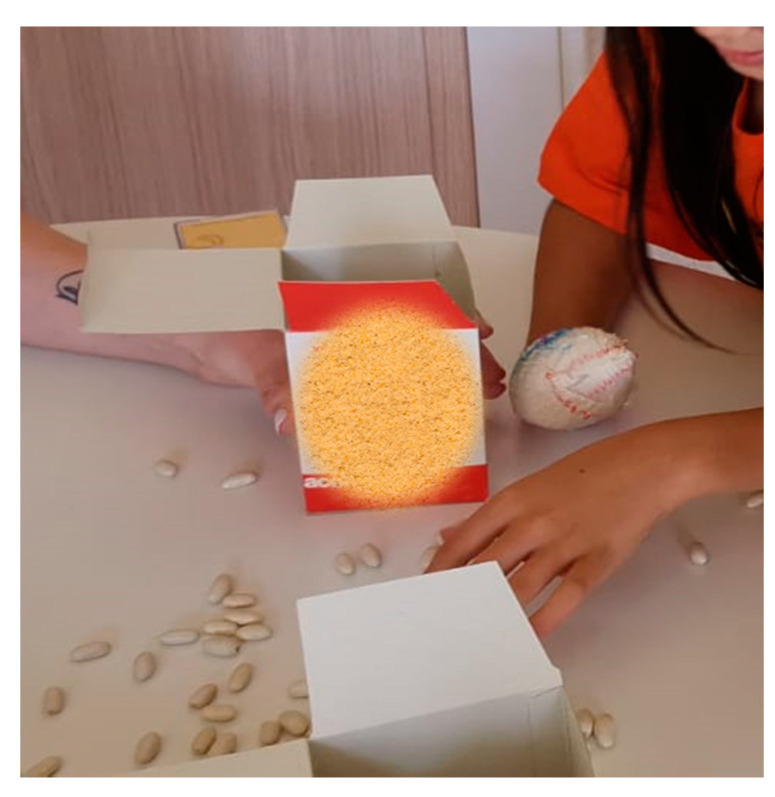
Child with left hemiplegia wearing a bandage as a partial containment in the right hand (dominant hand). In this task, the child is working the grasp–release action.

The containment applied was partial, such as a glove or a bandage [21]. In this way, manipulation with the healthy hand was prevented and the wrist and elbow joints were also free to allow the child to react effectively to an external disturbance (Figure 1).

Each week, the therapist and the parents of the participants had a meeting to follow-up the tasks and solve problems. The follow-up with the families was conducted online, reviewing all the activities and modifying those that were too difficult for the child, maintaining great therapist–family–child feedback. The activities were designed according to the limitations of the children and with simplicity in order for the latter to be able to perform them. In the follow-up, the activities that were not adequate were valued and changed for others considering the interest of the child.

**Figure 2:**
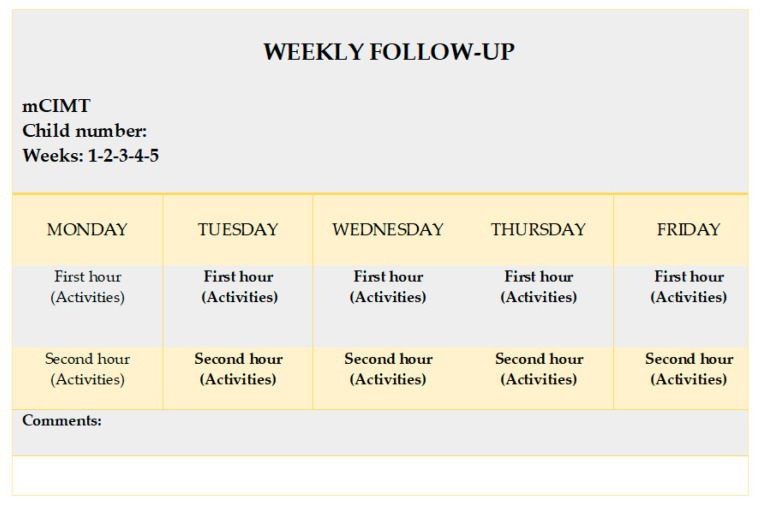
Weekly follow-up for the families.

The parents were asked to fill out a table with the performed tasks, completing these with photographs and videos of the tasks carried out daily and the execution time to complete 96% of total dose [13], in this case, 48 h (total dose: 50 h) (Figure 2).

### 2.2. Data Collection

**Figure 3:**
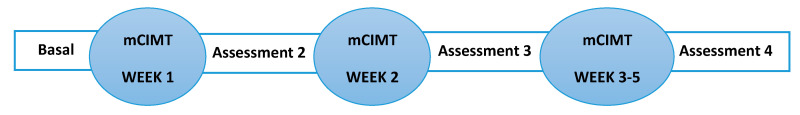
Representation of the assessment number in the 5 weeks of treatment (mCIMT).

Four assessments (Basal (T0, before therapy), T1 (Assessment 2, after Week 1 of therapy), T2 (Assessment 3, after Week 2 of therapy) and T3 (Assessment 4, after Week 5 of therapy)) were performed to measure the study variables and to compare the results before, during and after the intervention (Figure 3).

#### 2.2.1. Studied Variables and Measuring Instruments

##### Quality of Movement and Bimanual Dexterity of Both Upper Extremities

The quality of upper extremity test (QUEST) [38,39,40] was used, validated for children with neuromotor dysfunction with spasticity from 18 months to 8 years of age. It provides a numerical value that is obtained from the mean of the percentages in 36 items distributed in four categories: dissociated movements, grasp, weight bearing and protective extension of both extremities. It takes a value from 0 to 100 and can be expressed in percentages (%).

##### Active Extension of the Wrist and Active Supination of the Forearm in the Affected Upper Limb

Both variables were measured with an arm goniometer [41], making three measurements for each variable and selecting the best result. The measurements were made with the child sitting. Wrist extension was measured with elbow flexion, with the child leaning on a table to decrease muscle tension and associated reactions. Supination was measured with the forearm close to the body, avoiding trunk compensations to gain greater joint width.

##### Grasp Strength in the Affected Hand

**Figure 4:**
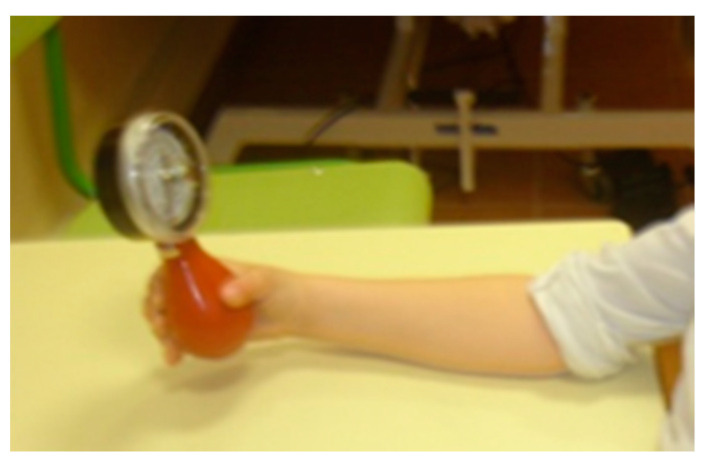
Grasp strength measurement with a hand dynamometer.

This was measured using a hand dynamometer [18] with a scale between 0 and 150 that expressed grasp strength in PSI (pound per square inch, 1 psi = 0.0703 kg/cm^2^). The test was performed with the child sitting on a chair in front of a table with the healthy hand under the table and the affected forearm resting on the table to give stability to the upper limb (Figure 4). The child was asked to press the dynamometer with the affected hand (global grasp) as hard as possible to obtain the best measurement.

##### Spontaneous Use, Dynamic Positioning of the Affected Upper Limb, Grasping and Releasing Action (Wrist Position in Neutral Flexion–Extension), and Level of Functionality and Integration of the Affected Upper Limb in Various Activities of Daily Living

The Shriners Hospital for Children Upper Extremity Evaluation (SHUEE) [40,42] was used to obtain the values in the four measurements. This evaluation consists of videotaping the children while they execute a series of tasks to observe the functionality and the joint alignment of the affected upper limb, and it has been validated for use in children with hemiplegia aged between 3 and 18 years. The results are expressed in percentages, with 100% being the best result.

The level of functionality and participation of the patient’s upper limbs was determined through the SHUEE assessment as dependent, assisted or independent. Activities of daily living, such as dressing upper limbs, dressing lower limbs, buttoning, putting on socks, putting on shoes, putting on splints and personal hygiene were assessed.

### 2.3. Statistical Analysis

Statistical analyses were performed using [43]. Given the small sample size, we used non-parametric tests. First, a Friedman’s test was used to evaluate the existence of statistical significance for the assessments performed at different times in each variable. Subsequently, a Wilcoxon test (paired samples) was performed on those variables that presented statistical significance, in order to observe statistically significant differences between paired assessments. Significance was set at a *p*-value of 0.05. The results are shown as medians and interquartile ranges (IQRs) with 95% confidence intervals. Those with a *p*-value < 0.05 were considered as significant values. The qualitative variable of “functionality” was turned into a quantitative variable, graduating it in 5 levels, from 0 = worst functionality to 4 = maximum functionality (Table 2).

## 3. Results

**Figure 5:**
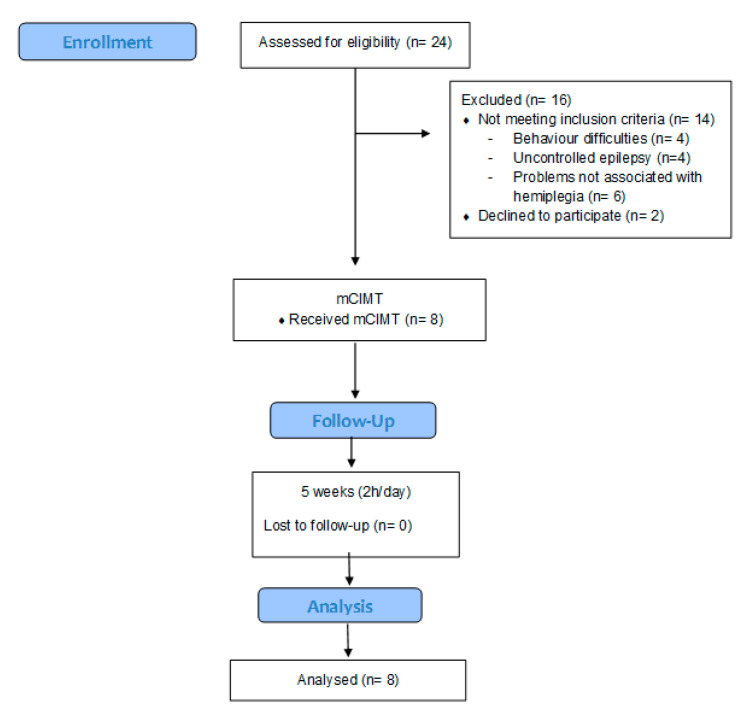
Flowchart of the sample selection.

Twenty-four children were recruited; 14 of them did not meet the inclusion criteria and two of them declined to participate. The final sample consisted of eight children (Figure 5), with 50% males and 50% females, diagnosed with congenital hemiplegia.

Of the entire sample, 62.5% had affectation of the left upper limb. The average age was 6 years, with a standard deviation of 1.77 years. After assessing the motor ability of the children, they were classified as Level I in the Gross Motor Function Classification System (GMFCS) [44] and Level II in the manual ability classification system (MACS) [34]. (Table 3)

### 3.1. Quality of Movement of the Upper Limb

The total score for the quality of movement in the upper limbs exhibited an increase of 94.07%. All the variables that compose it, i.e., dissociated movements, grasp, weight bearing and protective extension, showed statistical significance (*p* ≤ 0.001) in the Friedman test. When the Wilcoxon test was applied, dissociated movements and grasp obtained significant changes for all pairwise comparisons with a *p*-value < 0.03. However, no statistically significant differences were found for weight bearing between the second and third assessments (*p* = 0.14), second and fourth assessments (*p* = 0.10) or third and fourth assessments (*p* = 0.11), nor for protective extension between the baseline and second assessments (*p* = 0.07) or second and third assessments (*p* = 0.07). The results are shown in Table 4.

### 3.2. Grasp Strength

The Friedman test showed significance in all assessments (*p* < 0.001). All pairwise comparisons between assessments were statistically significant (*p* < 0.05), except between the baseline and second assessments (*p* = 1) (Table 5). The largest increase observed occurred from the third to the fourth measurement, with 1 PSI.

### 3.3. Active Elbow Extension and Forearm Supination

The Friedman test showed significance in all assessments (*p* < 0.001). Both variables increased their value in each of the assessments carried out, exhibiting an increase of 21° for elbow extension between the baseline and fourth measurements (*p* = 0.011), and an increase of 11.50° for the supination of the forearm (*p* = 0.011) between the baseline and fourth measurements (Table 6).

### 3.4. Spontaneous Use, Dynamic Joint Position of the Affected Upper Limb, Grasp and Release Action (Wrist Position in Neutral Flexion–Extension), and Level of Functionality and Integration of the Affected Upper Limb in Different Activities of Daily Living

Spontaneous use increased in all evaluations, reaching 88.87% in the fourth assessment, as did dynamic joint position and grasp–release action with different wrist positions, with 88.20% and 91.67%, respectively. These three variables showed statistical significance in the Friedman test with a *p*-value < 0.001. The pairwise comparison showed that, in spontaneous use, the values of the second and third assessments were not significant (*p* > 0.05) when compared with the values of the fourth measurement. In dynamic joint positioning, there were no significant differences between the second and third assessments (*p* = 0.237). Grasp–release action was only significant between the baseline and fourth assessments (*p* = 0.03) and between the second and fourth assessments (*p* = 0.04). (Table 7).

An increase was observed in all the variables of functionality and integration of the affected upper limb in various activities of daily living (proposed in SHUEE). All the increases were statistically significant (*p* < 0.05) in the Friedman test, except for “buttoning buttons”, where *p* = 0.163. In the pairwise comparison of the assessments for the action of dressing the upper limbs, putting on the splints and buttoning buttons, no statistical significance was detected (*p* > 0.05). In the action of dressing the lower limbs, statistically significant differences were obtained for all assessments, except between the baseline and second assessments and between the second and third assessments (*p* ≥ 0.05). In the action of putting on shoes, significant differences were not found between the baseline and second assessments (*p* = 0.32), between the baseline and fourth assessments (*p* = 0.06) or between the third and fourth assessments (*p* = 0.10). In the action of putting on socks, there were no differences between the baseline and second assessments (*p* = 1.00) or between the third and fourth assessments (*p* = 0.08). Regarding personal hygiene, only the changes between the baseline and fourth assessments and between the second and fourth assessments were significant (*p* = 0.02) (Table 8).

After analyzing the follow-up tables of the activities completed by each family, it was observed that five children performed the total dose (50 h), two children performed 48 h, and one of them performed 49 h. All of them completed the expected dose (48–50 h of mCIMT).

## 4. Discussion

The deterioration of hand functionality causes a weakness present in the execution of activities of daily living in children with hemiplegia. There is an alteration compared to the healthy upper limb that manifests in the general slowness of movement, discontinuous movements, variability in the trajectory of the hand with compensations of the trunk and the presence of inadequate coordination in the grasp strength of the affected hand [45]. The improvement in grasp strength and stability occurs from the third to the fourth measurement due to an increase in hand strength. The increase was observed only in the last measurement, which could be due to the need for a longer treatment time (5 weeks of intervention). These children with impaired fine motor adjustment, a lack of finger dissociation and deficient proprioception in their affected hand had greater experience (trial–error) to adapt the grasp to the shape, texture and weight of the object, allowing the execution of a previous thinking strategy (anticipatory control) to achieve precision in the grasp and adjustment of the strength to grasp the object adequately. The improvement of grasp stability and strength allows a functional grasp when picking up objects of different characteristics and holding them while performing selective and precision activities, such as throwing a small ball at a target, keeping a fork steady with the affected hand and bringing food to the mouth during the feeding phase. Most children with unilateral brain injury do not develop adequate grip strength in the affected upper limb to coordinate one-handed activities. There is a pathological pattern or an immature state of grasp for their age, leading to an inadequate synergy of the coordination strength that is related to the deterioration of the manual ability of the affected hand depending on the level of injury [46].

Children obtained significant changes in the functional activities of daily life assessed, except in dressing the upper limbs, putting on splints and buttoning buttons. This could suggest the need for improvements in visuomotor coordination and bimanual coordination, and greater strength and precision in the affected grasp to support objects and to perform the activities, which require great ability in the affected upper limb.

There are few studies that propose a home-based therapy intervention [12,13,17,19,25]. Among such studies, only three of them combine this proposal with a modification based on the application of low doses of treatment [12,19,25]. None of them were developed in Spain; thus, our preliminary results provide interesting and unpublished data of the application of mCIMT.

The advantage of combining a low dose of treatment with the application of therapy in the child’s own home is that this modification is better accepted by both parents and the child, as reported by authors such as Eliasson et al. [12], who showed better rates of parental competence among those who had applied low doses of treatment. Likewise, some children showed higher levels of frustration or low tolerance was shown by both the child and the family with higher doses of treatment. To minimize such feelings, some authors have proposed adapting the original protocol, suggesting the use of the containment only during the intervention period, reducing the dose and using a protocol that is “child friendly” and enhances children’s engagement [22,40,47]. Thus, our proposal could positively affect these aspects.

Although the objective of this study was not to analyze the cost–benefit of mCIMT, the positive results obtained in it demonstrate that this type of intervention is also a low-cost treatment compared to the application of botulinum toxin [11,14,48,49,50].

On the other hand, the age of the participants is also an important aspect to take into account when applying this treatment modality. The results of our study coincide with those of Chen et al. (2016), since the younger children with cerebral palsy responded better to home-based CIMT on some areas of upper limb functions than older children. When the child does not receive treatment, the choice of using the upper limbs to carry out a unimanual action will depend on the characteristics of the injury, levels of disability, experience and level of frustration and motivation in carrying out activities, among other factors. Learning “not to use” the unaffected upper limb by means of mCIMT intervention can provide an increased spontaneous participation of the affected upper limb in unimanual and bimanual tasks [32], observed in all measurements performed by SHUEE evaluation. The greatest increase was observed from the first to the second measurement, reaching 15.56% of the total value obtained in the last measurement, 18.87% being the median. This suggests that, when children do not depend on their dominant hand, they learn to use their affected upper limb early and acquire a greater representation within their body schema, developing functional strategies for the execution of daily tasks that allow them to overcome the “disuse” of the affected upper limb due to a lack of integration. Spontaneous use continued to evolve throughout the intervention as the children overcame the lack of experience of use. The improvement in functional performance was reflected in activities of daily living, where a degree of independence and greater participation of the affected limb was reached in the last measurement for the execution of the tasks of dressing upper limbs and lower limbs; putting on socks, shoes and splints; and personal hygiene. The activity carried out with the greatest ability and participation of both upper limbs was putting on pants, for which the most significant statistical results were obtained in the pairwise comparison of measurements. The increase in participation of the affected limb observed from the first measurement to the last, and in some activities accentuated in the fourth measurement, was due to an increase in the quality of bimanual coordination, as a result of the greater integration of the affected upper limb. The parents of the participants provided information about the use of the affected limb in their usual environment, such as during meals to actively support the healthy limb, without the need for the parents to give a verbal order to the child to use it; execution of school activities; playing (symbolic play with dolls); and in extracurricular activities such as dancing, where greater integration and earlier activation of the affected segment was observed, among others, which allowed reducing frustration and abandoning the disuse of the affected upper limb. The families showed great satisfaction with this protocol, which was reflected in the adherence to the therapy, since the children completed 96% of the total dose, thus suggesting the importance of the family and the setting. The natural environment (home) offered less distress during constraint-induced movement therapy practice for both children with cerebral palsy and their parents. Furthermore, the training schedule can be tailored to fit the family’s daily routine. A home-based intervention can also save the family time and money in terms of commuting, and parents can be more involved throughout the process, increasing opportunities for parent–child interaction [51]. There could be a continuity in the maintenance of the gains obtained in the functionality of the affected upper limb after executing the intervention, since the daily treatment session was carried out at home, simulating the situation of normal life for the child, in addition to the design of the treatment protocol to simulate activities of daily living. The improvement in the quality of movement was shown by the greater progress in the results obtained between the baseline and fourth measurements of the variables of dissociated movements and grasp. Appreciable benefits were obtained in each measurement produced by the acquisition of a more corrected posture of the trunk, head and shoulders in the execution of the grasp activities, present from the second measurement (after a week of treatment with mCIMT). A dynamic joint position occurred in wrist and elbow extension, increasing the median value for wrist extension by 21° from the baseline to the fourth measurement. In addition, the value reached in the fourth measurement for the median was 81.50° in the active supination of the forearm, allowing for greater control and support of the body structures for the execution of dissociated movements, grasp, weight bearing and protective extension due to the improvement of both active movements. There was a favorable evolution in the dynamic joint position of the affected upper limb due to the gain of active degrees for movement restriction in extension and supination, with an increase in this variable of 10.42% at the end of the treatment. In this way, the activities proposed during the evaluation were performed with greater ease of movement, such as eating a cookie, touching the opposite ear, picking up coins, opening a bottle or throwing a large ball, which require the selective motor control of certain muscles. In comparison with the results obtained for the quality of movement of the affected upper limb in the present study, we highlight an investigation [52] on mCIMT, which showed positive results for the assessment of the quality of movement of motor skills (measured through the QUEST scale) using an intervention protocol of 3 weeks of treatment with an intensity of 6 h per day of restriction and repetitive work. This study demonstrated the effectiveness of the intervention, as it had a larger sample and a control group (18 children with hemiplegia, nine children in the experimental group and nine children in the control group). In 2011, a different study [53], conducted exclusively with a girl with hemiplegia, used mCIMT for one hour per day for two weeks. No significant results were obtained at the end of the treatment, which was thereby prolonged for one more week of intervention. This last assessment showed an increase in the overall percentage of total quality of movement (also measured by the QUEST scale), appreciable in activities that involve the affected upper limb. Thus, the protocol chosen for the intervention and the initial functionality of the patient are two important factors to take into account in increasing the results obtained in the measurements. When comparing the results obtained in the second assessment after 20 h of treatment with those obtained after the treatment (50 h), improvements were detected in the former (20 h), although the greatest improvements and relevant changes were identified in the latter (50 h). At the end of the treatment, the families observed these functional changes (daily activities assessed) at different natural moments. Thus, it would be necessary to apply 50 h of dose instead of 20 h in children aged 4–8 years to increase the affected upper limb functionality, since the manual ability and age would be factors to consider in the dose to apply.

### Limitations and Future Lines of Research

As this is an uncontrolled trial [54] due to the absence of a control group, it cannot be guaranteed that the observed response (changes produced throughout the intervention with respect to the baseline situation) is exclusively due to the mCIMT protocol used, since other uncontrolled factors may also have had an influence on it. Therefore, the effectiveness of the mCIMT in increasing the functionality of the affected upper limb seen in the present study cannot be generalized to the population of children with hemiplegia, and thus, the fundamental utility of this study is descriptive. It is important to highlight the statistically significant results obtained in the different studied variables throughout the 5 weeks of the intervention (It can be seen at Figures S1–S11. Progression of different variables in the four assessments over 5 weeks of mCIMT). This suggests that, although there was no control group, it could be inferred that the changes obtained in the improvement of the functionality were due to the efficacy of the treatment, since it was a short period of time, where important differences were observed in the affected upper limb, which is unlikely to occur due to the maturation effect (learning and natural development of the child over time).

In Spain, this is the first study to provide preliminary data on the use of mCIMT. Although the results should be interpreted with caution due to the small sample size, the development of this pilot scheme is the first step for this type of therapy, which has already demonstrated its effectiveness in other contexts; therefore, it should be taken into account by therapist and researchers who develop their work with this type of children in Spain.

This study leads us to open different lines of research, such as including a control group to assess the effectiveness of the treatment in the functionality of the affected upper limb, to verify whether the gains obtained after the intervention are maintained over time (6 months or one year after therapy), and to study the influence of age on the obtained results due to neuronal plasticity and active participation of the subject. Likewise, it paves the road for clinicians and researchers to develop new treatment proposals in Spain.

## 5. Conclusions

A low dose (50 h) of modified Constraint-Induced Movement Therapy can increase the functionality of children diagnosed with congenital hemiplegia between 4 and 8 years of age with moderate manual ability.

## Figures and Tables

**Table 1:** Example of “modified Constraint-Induced Movement Therapy” (mCIMT) designed tasks.

Movements to Work in the Affected Upper Limb	Examples of mCIMT Designed Tasks
Shoulder flexion and elbow extension	Put stickers at different heights on the wall and try to cover them with the affected hand. Take a small and light ball and try to throw it higher towards a target. The parents will put a cardboard or continuous paper stuck on the wall; using finger paint, the child will try to draw a picture or put his/her hand with paint on the paper. The parents will throw balloons or bubbles, which the child will try to hit with his/her hand. The parents will give the child a small and light ball, which he/she will try to throw higher and higher or towards a target.
Forearm supination	Put stickers on the palm of the affected hand or forearm. Fishing game. The parents will place a light object on the palm of the affected hand (for example, a colored pompom) and the child will keep it for a while. The child will use the affected hand to remove objects stuck on his/her t-shirt (at the level of the abdomen). Playing the trumpet or other instruments…
Wrist extension	The parents will push cardboard boxes or other elements and the child will try to throw them off the table. The child will smash packing paper, balls and/or soft objects with the palm of his/her hand. The child must roll a ball, bottle. The child must hit a piano or a drum placed vertically. The child will remove pieces fallen from the wall.
Grasp–release	Grasp, hold and transfer light and long objects. Grasp, hold and transfer heavy, long and rough objects. Grasp, hold and transfer rough, light and spherical objects. Grasp, hold and transfer rough, heavy and spherical objects. Grasp, hold and transfer different objects with a variety of the previous characteristics.

**Table 2:** Description of the functionality levels.

**Dependent**	Value 0. Needs help from an adult (does not perform the action).
**Assisted**	Value 1. Needs help from an adult (partially performs the action).
**Independent**	Value 2. Uses the healthy upper limb exclusively.
	Value 3. Uses the upper limb to provide stability.
	Value 4. Uses both upper limbs to execute the action.

**Table 3:** Baseline characteristics of the participants.

Sample	Age	Sex	Gest. Week	Lesion	MACS Level	GMFCS Level	Hemiplegia Side	Add. Impair
Child 1	4	F	Term	Perinatal stroke	II	I	Left	No
Child 2	5	M	Term	Perinatal stroke	II	I	Right	No
Child 3	5	M	Preterm	Perinatal stroke	II	I	Left	No
Child 4	5	M	Preterm	Perinatal stroke	II	I	Left	No
Child 5	7	F	Term	Perinatal stroke	II	I	Right	Speech
Child 6	6	F	Term	Perinatal stroke	II	I	Right	Speech
Child 7	8	F	Preterm	Perinatal stroke	II	I	Left	Speech
Child 8	8	M	Term	Perinatal stroke	II	I	Left	No

Sex: F: Female; M: Male. Gest. Week: Gestational Week: preterm (>32 weeks and <36 weeks); term: >36 weeks; MACS: Manual Ability Classification System; GMFCS: Gross Motor Function Classification System. Add. Impair: Added impairment.

**Table 4:** Results and pairwise comparisons for the quality of movement in the upper limbs.

Variables	Results	Friedman’s Test		Wilcoxon’s Test		
	Median (IQR)	Statistical Significance	*p* Value	Pairwise Comparisons	Statistical Significance	*p* Value
Quality of Movement in the Upper Limbs (Total Score)						
Baseline	74.16 (63.55, 83.00)	24.000	0.000 *	Baseline–2nd assessment	−2.521	0.012 *
2nd assessment	83.32 (77.18, 88.49)			Baseline–3rd assessment	−2.521	0.012 *
3rd assessment	88.70 (84.42, 91.42)			Baseline–4th assessment	−2.521	0.012 *
4th assessment	94.07 (90.32, 94.92)			2nd assessment–3rd assessment	−2.521	0.012 *
				2nd assessment–4th assessment	−2.521	0.012 *
				3rd assessment–4th assessment	−2.521	0.012 *
Dissociated Movements						
Baseline	59.38 (53.90, 80.48)	23.538	0.000 *	Baseline–2nd assessment	−2.524	0.012 *
2nd assessment	75.00 (73.83, 82.82)			Baseline–3rd assessment	−2.521	0.012 *
3rd assessment	83.60 (81.24, 87.11)			Baseline–4th assessment	−2.521	0.012 *
4th assessment	89.84 (84.77, 91.79)			2nd assessment–3rd assessment	−2.201	0.028 *
				2nd assessment–4th assessment	−2.524	0.012 *
				3rd assessment–4th assessment	−2.527	0.012 *
Grasp						
Baseline	62.97 (46.30, 76.86)	21.808	0.000 *	Baseline–2nd assessment	−2.533	0.011 *
2nd assessment	79.60 (59.26, 87.96)			Baseline–3rd assessment	−2.527	0.012 *
3rd assessment	87.03 (63.89, 91.67)			Baseline–4th assessment	−2.527	0.012 *
4th assessment	96.30 (75.00, 96.30)			2nd assessment–3rd assessment	−2.200	0.028 *
				2nd assessment–4th assessment	−2.384	0.017 *
				3rd assessment–4th assessment	−2.384	0.017 *
Weight Bearing						
Baseline	87.00 (76.50, 93.50)	19.154	0.000 *	Baseline–2nd assessment	−2.536	0.011 *
2nd assessment	98.00 (85.50, 99.50)			Baseline–3rd assessment	−2.524	0.012 *
3rd assessment	97.00 (93.00, 99.50)			Baseline–4th assessment	−2.521	0.012 *
4th assessment	99.00 (96.00, 100.00)			2nd assessment–3rd assessment	−1.461	0.144
				2nd assessment–4th assessment	−1.461	0.102
				3rd assessment–4th assessment	−1.604	0.109
Protective Extension						
Baseline	80.56 (75.00, 90.27)	17.431	0.001 *	Baseline–2nd assessment	−1.841	0.066
2nd assessment	83.34 (79.17, 96.53)			Baseline–3rd assessment	−2.207	0.027 *
3rd assessment	90.27 (84.73, 97.92)			Baseline–4th assessment	−2.379	0.017 *
4th assessment	94.44 (92.36, 99.31)			2nd assessment–3rd assessment	−1.826	0.068
				2nd assessment–4th assessment	−2.207	0.027 *
				3rd assessment–4th assessment	−2.023	0.043 *

* Statistically significant when the *p*-value < 0.05; quality of movement measured with quality of upper extremity test (QUEST) scale. Results expressed in percentages (%) as median (IQR).

**Table 5:** Results and pairwise comparisons of grasp strength.

Variable: Grasp Strength	Results	Friedman Test		Wilcoxon Test		
	Median (IQR)	Statistical Significance	*p* Value	Pairwise Comparisons	Statistical Significance	*p* Value
Baseline	2.00 (1.00, 2.75)	20.069	0.000 *	Baseline–2nd assessment	0.000	1.000
2nd assessment	2.00 (1.00, 2.75)			Baseline–3rd assessment	−2.000	0.046 *
3rd assessment	2.00 (1.25, 3.75)			Baseline–4th assessment	−2.640	0.008 *
4th assessment	3.00 (2.25, 4.50)			2nd assessment–3rd assessment	−2.000	0.046 *
				2nd assessment–4th assessment	−2.640	0.008 *
				3rd assessment–4th assessment	−2.449	0.014 *

* Statistically significant when *p* value < 0.05. Results expressed in median (IQR) measured in PSI units.

**Table 6:** Results and pairwise comparisons of elbow extension and forearm supination.

Variables: Active Elbow Extension	Results	Friedman Test		Wilcoxon Test		
	Median (IQR)	Statistical Significance	*p* Value	Pairwise Comparisons	Statistical	*p* Value
Baseline	12.50 (10.00, 43.75)	23.423	0.000 *	Baseline–2nd assessment	−2.565	0.010 *
2nd assessment	22.50 (20.00, 50.00)			Baseline–3rd assessment	−2.533	0.011 *
3rd assessment	27.50 (22.75, 54.50)			Baseline–4th assessment	−2.536	0.011 *
4th assessment	33.50 (25.75, 64.25)			2nd Assessment–3rd assessment	−2.456	0.014 *
				2nd assessment–4th assessment	−2.536	0.011 *
				3rd assessment–4th assessment	−2.375	0.018 *
Active Forearm Supination						
Baseline	70.00 (35.50, 75.00)	23.416	0.000 *	Baseline–2nd assessment	−2.588	0.010 *
2nd assessment	75.00 (45.00, 80.00)			Baseline–3rd assessment	−2.536	0.011 *
3rd assessment	76.50 (53.50, 82.25)			Baseline–4th assessment	−2.524	0.012 *
4th assessment	81.50 (58.75, 87.75)			2nd Assessment–3rd assessment	−2.032	0.042 *
				2nd assessment–4th assessment	−2.536	0.011 *
				3rd assessment–4th assessment	−2.527	0.012 *

* Statistically significant when *p* < 0.05. Results expressed in median (IQR), measured in degrees of movement.

**Table 7:** Results and pairwise comparisons of spontaneous use, dynamic joint position and grasp–release action.

Variable	Results	Friedman’s Test		Wilcoxon’s Test		
Spontaneous Use in the Affected Upper Limb	Median (IQR)	Statistical Significance	*p* Value	Pairwise Comparisons	Statistical Significance	*p* Value
Baseline	70.00 (49.45, 87.78)	18.932	0.000 *	Baseline–2nd assessment	−2.371	0.018 *
2nd assessment	85.56 (58.34, 95.00)			Baseline–3rd assessment	−2.366	0.018 *
3rd assessment	87.78 (72.78, 95.55)			Baseline–4th assessment	−2.521	0.012 *
4th assessment	88.87 (87.11, 97.22)			2nd assessment–3rd assessment	−2.201	0.028 *
				2nd assessment–4th assessment	−1.963	0.050
				3rd assessment–4th assessment	−1.690	0.091
Dynamic Joint Position						
Baseline	77.78 (48.24, 86.81)	23.416	0.000 *	Baseline–2nd assessment	−2.366	0.018 *
2nd assessment	80.56 (75.35, 89.93)			Baseline–3rd assessment	−2.521	0.012 *
3rd assessment	85.44 (71.87, 9132)			Baseline–4th assessment	−2.521	0.012 *
4th assessment	88.20 (84.03, 92.71)			2nd assessment–3rd assessment	−1.183	0.237
				2nd assessment–4th assessment	−2.366	0.018 *
				3rd assessment–4th assessment	−2.023	0.043 *
Grasp–Release action						
Baseline	58.34 (50.00, 91.67)	13.568	0.004 *	Baseline–2nd assessment	−1.414	0.157
2nd assessment	75.00 (54.17, 95.83)			Baseline–3rd assessment	−1.890	0.059
3rd assessment	83.33 (66.67, 100.00)			Baseline–4th assessment	−2.226	0.026 *
4th assessment	91.67 (83.33, 100.00)			2nd assessment–3rd assessment	−1.342	0.180
				2nd assessment–4th assessment	−2.041	0.041 *
				3rd assessment–4th assessment	−1.857	0.063

* Statistically significant when *p* < 0.05. Results expressed in percentages % as median (IQR) measured with SHUEE.

**Table 8:** Results and pairwise comparisons of upper limb participation in activities of daily living (SHUEE).

Variable	Results	Friedman’s Test		Wilcoxon’s Test		
Upper Limb Dressing	Median (IQR)	Statistical Significance	*p* Value	Pairwise Comparisons	Statistical Significance	*p* Value
Baseline	3.50 (2.25, 4.00)	10.355	0.016 *	Baseline–2nd assessment	0.000	1.000
2nd assessment	3.50 (2.25, 4.00)			Baseline–3rd assessment	−1.633	0.102
3rd assessment	4.00 (3.25, 4.00)			Baseline–4th assessment	−1.857	0.063
4th assessment	4.00 (4.00, 4.00)			2nd assessment–3rd assessment	−1.633	0.102
				2nd assessment–4th assessment	−1.857	0.063
				3rd assessment–4th assessment	−1.414	0.157
Lower Limb Dressing						
Baseline	3.00 (2.25, 3.00)	15.245	0.002 *	Baseline–2nd assessment	−1.414	0.157
2nd assessment	3.00 (3.00, 3.75)			Baseline–3rd assessment	−2.000	0.046 *
3rd assessment	3.00 (3.00, 4.00)			Baseline–4th assessment	−2.530	0.011 *
4th assessment	4.00 (4.00, 4.00)			2nd assessment–3rd assessment	−1.414	0.157
				2nd assessment–4th assessment	−2.449	0.014 *
				3rd assessment–4th assessment	−2.000	0.046 *
Putting on Splints						
Baseline	3.00 (1.00, 3.75)	9.923	0.019 *	Baseline–2nd assessment	0.000	1.000
2nd assessment	3.00 (1.00, 3.75)			Baseline–3rd assessment	−1.633	0.102
3rd assessment	3.00 (1.00, 4.00)			Baseline–4th assessment	−1.857	0.063
4th assessment	4.00 (1.50, 4.00)			2nd assessment–3rd assessment	−1.633	0.102
				2nd assessment–4th assessment	−1.857	0.063
				3rd assessment–4th assessment	−1.414	0.157
Putting on Shoes						
Baseline	2.00 (2.00, 3.50)	15.188	0.002 *	Baseline–2nd assessment	−1.000	0.317
2nd assessment	2.00 (2.00, 3.75)			Baseline–3rd assessment	−2.121	0.034 *
3rd assessment	3.00 (3.00, 4.00)			Baseline–4th assessment	−2.271	0.023 *
4th assessment	4.00 (3.25, 4.00)			2nd assessment–3rd assessment	−1.890	0.059
				2nd assessment–4th assessment	−2.251	0.024 *
				3rd assessment–4th assessment	−1.633	0.102
Putting on Socks						
Baseline	3.00 (3.00, 3.75)	15.000	0.002 *	Baseline–2nd assessment	0.000	1.000
2nd assessment	3.00 (3.00, 3.75)			Baseline–3rd assessment	−2.000	0.046 *
3rd assessment	3.00 (3.00, 4.00)			Baseline–4th assessment	−2.333	0.020 *
4th assessment	4.00 (4.00, 4.00)			2nd assessment–3rd assessment	−2.000	0.046 *
				2nd assessment–4th assessment	−2.333	0.020 *
				3rd assessment–4th assessment	−1.732	0.083
Buttoning Up						
Baseline	1.00 (0.00, 3.00)	5.118	0.163	Baseline–2nd assessment	−1.000	0.317
2nd assessment	1.50 (0.00, 3.00)			Baseline–3rd assessment	−1.414	0.157
3rd assessment	1.50 (0.00, 3.75)			Baseline–4th assessment	−1.342	0.180
4th assessment	1.50 (0.00, 4.00)			2nd assessment–3rd assessment	−1.000	0.317
				2nd assessment–4th assessment	−1.414	0.157
				3rd assessment–4th assessment	−1.000	0.317
Personal Hygiene						
Baseline	2.00 (2.00, 2.00)	15.000	0.002 *	Baseline–2nd assessment	0.000	1.000
2nd assessment	2.00 (2.00, 2.00)			Baseline–3rd assessment	−1.890	0.059
3rd assessment	2.50 (2.00, 4.00)			Baseline–4th assessment	−2.333	0.020 *
4th assessment	4.00 (2.25, 4.00)			2nd assessment–3rd assessment	−1.890	0.059
				2nd assessment–4th assessment	−2.333	0.020 *
				3rd assessment–4th assessment	−1.633	0.102

* Statistically significant when *p* < 0.05. Results expressed in median (IQR), measured with SHUEE on a scale of 1 to 5.

## Supplementary Materials

The following are available online at https://www.mdpi.com/2227-9067/7/9/127/s1. Supplementary figures: Figures S1–S11. Progression of different variables in the four assessments over 5 weeks of mCIMT.
