# Supplementary material for: Application of Low-Intensity Modified Constraint-Induced Movement Therapy to Improve the Affected Upper Limb Functionality in Infantile Hemiplegia with Moderate Manual Ability: Case Series
Source: Children (Basel). 2020 Sep 4;7(9):127. doi: 10.3390/children7090127 (PMC7552787; doi:10.3390/children7090127)
Supplement: Supplementary file 1 [file children-07-00127-s001.docx]

|  |
| --- |
| **Figure S1.** Progression of elbow extension in the four assessments during the five weeks of treatment |

|  |
| --- |
| **Figure S2.** Progression of forearm supination in the four assessments during the five weeks of treatment |

|  |
| --- |
| **Figure S3.** Progression of strenght grasp in the four assessments during the five weeks of treatment |

|  |
| --- |
| **Figure S4**. Progression of spontaneous use in the four assessments during the five weeks of treatment |

|  |
| --- |
| **Figure S5.** Progression of dynamic position in the four assessments during the five weeks of treatment |

|  |
| --- |
| **Figure S6.** Progression of grasp-release action in the four assessments during the five weeks of treatment |

|  |
| --- |
| **Figure S7.** Progression of qualit of movement (total score) in the four assessments during the five weeks of treatment |
|  |
| **Figure S8.** Progression of dissociated movements in the four assessments during the five weeks of treatment |

|  |
| --- |
| **Figure S9.** Progression of grasp in the four assessments during the five weeks of treatment |

|  |
| --- |
| **Figure S10.** Progression of weight bearing in the four assessments during the five weeks of treatment |

|  |
| --- |
| **Figure S11.** Progression of protective extension in the four assessments during the five weeks of treatment |
